# Supplementary material for: Are carbon emissions trading and green financial instruments synergistic? -Comprehensive quantitative research based on content analysis
Source: PLoS One. 2024 Mar 7;19(3):e0298601. doi: 10.1371/journal.pone.0298601 (PMC10919601; doi:10.1371/journal.pone.0298601)
Supplement: S1 Data — (ZIP) [file pone.0298601.s001.zip › Data/地级市Z_F工作报告.zip!/╣π╢1⁄2/╖≡╔╜╩╨/└┤╫╘╣1⁄2╓┌║┼í╛┬φ┐╦╩2╛▌═°í┐_2018.docx]

各位代表：

　　现在，我代表市人民政府，向大会报告政府工作，请予审议，并请各位政协委员和其他列席人员提出意见。

2017年工作回顾

　　2017年是本届政府开局之年，是实施“十三五”规划的重要一年，也是供给侧结构性改革深化之年。市政府在省委、省政府和市委的正确领导下，在市人大、市政协的监督支持下，认真贯彻落实党的十八大、十九大精神，坚持以习近平总书记对广东工作重要指示批示精神为统领，不断增强“四个意识”，坚持稳中求进工作总基调，牢固树立新发展理念，以推进供给侧结构性改革为主线，砥砺奋进、真抓实干，努力做好稳增长、促改革、调结构、惠民生、防风险各项工作，全市经济社会保持平稳健康发展，顺利完成市第十五届人大一次会议确定的各项任务。预计实现地区生产总值9549.6亿元，增长8.5%；地方一般公共预算收入661.4亿元，增长11.6%；固定资产投资4265.8亿元，增长21.5%；社会消费品零售总额3320.4亿元，增长10%；进出口总额4357.4亿元，增长6.1%；居民人均可支配收入4.5万元，增速与经济增长基本同步；城镇登记失业率2.35%；居民消费价格涨幅1.9%；单位地区生产总值能耗、主要污染物排放量完成省下达的目标任务。

　　一年来，我们主要做了以下工作：

　　一是保持经济稳中向好。实施稳增长系列举措，经济运行高开稳走、稳中有进。工业生产持续加快。以稳工业促稳增长，工业生产延续2016年一季度以来持续加快走势，增幅创两年来新高，主引擎作用凸显。预计完成规模以上工业总产值2.24万亿元，增长8.7%；工业增加值4930.1亿元，增长8.8%。工业生产者出厂价格指数上涨4.5%；制造业采购经理指数持续走高，连续15个月处于荣枯线以上，12月达55%；工业用电量468.6亿千瓦时，增长8%，大工业实增容量增长32.9倍。工业结构持续优化，完成先进制造业总产值1.02万亿元，增长9.8%，占规模以上工业总产值比重45.8%，提高6.6个百分点；高技术制造业总产值1739.8亿元，增长16.3%。工业经济效益稳步向好，预计规模以上工业企业主营业务收入增长14.7%，提高7.1个百分点；主营业务收入利润率7.9%，提高0.14个百分点；亏损面8.8%，下降0.9个百分点。适度扩大总需求。出台扩大固定资产投资工作方案，多渠道促进有效投资增长。完成工业投资1690.7亿元，增长15.5%，总量超房地产开发投资236.7亿元，连续6年位居全省首位；基础设施投资829.7亿元，增长53.6%。实施扩内需促消费措施，创建省放心消费试点城市，发展型享受型消费增长较快，汽车类零售额651.7亿元，增长13.7%；快递业务收入增长30%。外贸保持平稳增长。完成出口总额3153.6亿元，增长1.7%；进口总额1203.8亿元，增长19.6%；一般贸易出口1818.1亿元，增长11%，占出口总额比重56.8%，提高3.8个百分点。民营经济活力增强。落实促进民营经济发展举措，着力维护民营企业及企业家合法经营权和产权，完善中小微企业综合服务体系，推动大型骨干企业跨越发展。深入开展“暖企”行动，走访调研企业1.1万家，收集意见建议并妥善解决。规模以上民营工业总产值1.58万亿元，增长10.2%，民营工业对全市工业增长贡献率81.6%；完成民间投资2984.4亿元，增长18.1%，占固定资产投资比重70%。新增主营业务收入超100亿元企业1家，总数17家。7家企业进入“2017中国民营企业500强”［1］。出台“四上”企业［2］培育三年行动计划，入库企业净增超1000家，总数1.1万家。

　　二是深化供给侧结构性改革。出台深化供给侧结构性改革实施意见，在全省率先设立5亿元改革专项基金，聚焦提升质量和降低成本。供给体系质量稳步提升。实施工业产品质量提升行动计划和扶持办法，创建全国质量强市示范城市通过省预验收。认定细分行业龙头企业154家；新增质量管理体系认证企业1525家，累计6642家；工业产品质量监督抽查综合合格率93.5％，提高1.5个百分点。新增联盟标准26项，累计98项；主导或参与制（修）订国家、行业或地方标准41项，累计1640项；新增采用国际标准认证产品89个，累计2592个。南海区成立全国首个制造业“隐形冠军”联盟。中国驰名商标159件，位居全国地级市首位；全国知名品牌示范区新申报4个，累计创建9个。弘扬企业家精神，表彰20家“佛山·脊梁企业”和20位“佛山·大城企业家”。“三去一降一补”取得实效。完成33户国有特困企业脱困出清，排查关停国有企业累计出清60户，超额完成考核目标。贯彻落实中央房地产调控政策，提前超额完成三年商品房去库存任务，累计净去化371.3万平方米，总去化周期9.5个月。积极构建租购并举长效机制，成立市建鑫住房租赁有限公司，向社会推出首批518套租赁住房。实施降低企业成本“佛十条”［3］等政策措施，帮助企业减负349.2亿元，其中新政策减负122.5亿元。57项补齐软硬基础设施短板重大项目进展顺利。推进农业供给侧结构性改革。积极培育壮大农业生产主体，新增省、市级现代农业园区16家，累计45家，入驻企业1199家；新增省级农业龙头企业12家，累计40家。实施耕地质量保护与提升行动，扩大绿色优质农产品供给，“三品一标一名牌”［4］认证产品累计224个，市级以上“菜篮子”基地66家。建设全省首个农业科技示范市。狠抓重点领域风险防控。规范政府举债融资和偿债机制，合理分配地方政府债券置换额度，优化债务结构，化解存量债务，预警地区债务风险水平总体下降。加强金融风险防控，清理整顿非法集资、互联网金融等重点领域，银行不良贷款率稳步下降，金融运行稳健，金融机构本外币存款余额1.4万亿元，增长5.7%；贷款余额0.94万亿元，增长7.6%。

　　三是推动制造业转型升级。实施工业转型升级攻坚战，促进存量优化、增量优质，推动制造业迈向中高端。优化升级传统产业。坚持以技术改造、智能化高端化、拥抱互联网等促进传统优势产业全产业链跃升。完成工业技术改造投资771.5亿元，增长39.4%，连续4年总量位居全省首位，累计超过一半规模以上工业企业开展技术改造。实施“机器人引领”行动，新增市级“中国制造2025”试点示范企业29家、智能化技改示范企业27家、机器人应用2227台。积极引导传统产业集群资源整合，推动搭建“众塑联”平台。发挥龙头带动作用推动珠江西岸先进装备制造产业带建设，成功举办第三届珠江西岸先进装备制造业投资贸易洽谈会。装备制造业增加值1654.5亿元，增长13.5%，其中“工作母机”［5］增加值347.4亿元，增长14.8%。深入实施“互联网+”行动计划，圆满举办第三届中国（广东）国际“互联网+”博览会。新增国家级两化融合管理体系贯标试点企业2家，累计21家；　新增省级19家，累计112家。积极培育新兴产业。落实军民融合发展战略，建设佛山军民融合创新示范区。发展机器人产业，省战略性新兴产业智能制造骨干（培育）企业达28家、省机器人骨干（培育）企业达7家。以招商引资促增量优质。组建市投资促进中心、佛山（深圳）产业联络处，设立招商引资重大项目专项资金，制定招商引资行为指引。签约投资额超亿元内资项目395个，计划投资3016.3亿元；超千万美元外资项目46个，计划投资35亿美元。引进达安创谷、新能源汽车小镇等平台项目，长江氢动力、巴斯巴、天劲等工业项目，宋城·西樵山、东方动漫等文旅项目，东丽等外资项目［6］。推动制造业与服务业融合发展。新增省级工业设计企业5家，累计10家；广东工业设计城聚集设计研发人员超8000名。国家物流标准化试点城市建设加快，2家企业入选商务部年度电子商务示范企业。广东潭洲国际会展中心全年举办展会活动19场次，二期建设加快。

　　四是实施创新驱动发展战略。加大科技创新投入，预计全社会研发经费占地区生产总值比重超2.7%。全面落实省创新驱动“八大举措”。积极培育高新技术企业，新增国家高新技术企业1173家，累计2561家。支持企业建立研发机构，规模以上工业企业研发机构建有率45%，其中主营业务收入5亿元以上工业企业实现全覆盖；新增省企业重点实验室4家，累计21家。启动建设先进制造科学与技术广东省实验室。强化政产学研用协同创新。成功举办第三届广东院士高峰年会、第22届全国发明展览会暨第二届世界发明创新论坛、佛山企业走进清华等活动。与省科学院签订战略合作协议，引进广东国防科技工业技术成果产业化应用推广中心、佛山中国空间技术研究院创新中心等平台。新增孵化器10家，累计62家；新增众创空间25家，累计57家。其中，国家级孵化器14家、国家级众创空间15家。加强人才培育引进，新增市级创新团队19个，累计76个。出台加快高等教育发展实施意见，分别与南方医科大学、广东财经大学共建全学段佛山校区，与北京科技大学、北京外国语大学共建佛山研究生院，创建2个研究生联合培养国家示范基地。佛山科学技术学院创建高水平理工科大学加快推进，仙溪新校区建成招生。推动金融科技产业融合发展，新增上市企业12家，累计54家，上市企业市值位列全国第7名［7］。新增新三板挂牌企业27家，累计100家；新增融资租赁公司4家，累计23家；新增私募股权投资基金52家，累计386家。成立规模1.25亿元的债券融资风险缓释基金。中国（佛山）知识产权保护中心基本建成。提升高端创新载体集聚能力。推进珠三角国家自主创新示范区建设，启动“一环创新圈”和禅南顺（三龙湾）高端创新集聚区规划，主动对接广深科技创新走廊。佛山国家高新区全国排名由第38位上升至第29位。广东金融高新区累计引进项目369个，总投资额近700亿元。

　　五是提升城市治理水平。实施城市治理三年行动计划，968个项目已完工137个、开工706个，累计完成投资额1161.3亿元。城市基础设施建设扎实推进。珠三角新干线机场启动前期工作，佛山西站开通运营，地铁2号线一期、3号线、广州地铁7号线西延顺德段、南海新交通试验线、高明有轨电车示范线建设稳步推进，“一环”高速化改造加快，“一环”西拓工程全面开工，新打通20条“断头路”，建成中心城区“五纵六横”公交骨干线网。启动海绵城市建设项目121个，建成地下综合管廊11.7公里。新建4G基站1.4万座，累计7.7万座；新增光纤接入用户超40万户，累计超230万户。镇村现代化建设步伐加快。规划建设特色小镇，创建首批15个市级特色小镇［8］，北滘智造小镇、西樵岭南文旅小镇、乐从国际品牌小镇入选国家级特色小镇，陶谷小镇、千灯湖创投小镇、特色小镇集群示范区（北滘—龙江—乐从—陈村）入选省特色小镇创建工作示范点。实施百村升级扩面计划，新启动20个特色古村落活化，推进88个城中村和旧社区改造、115个“五好”新村居和110个美丽文明示范村居建设。完成749个村（居）“两委”换届选举。南海区和南庄镇龙津村入选首批全国农村幸福社区建设示范单位。生态环境治理力度加大。创建成为国家森林城市，编制《自然生态文明建设专项规划》，建成区绿化覆盖率42.8%。全面推行河长制，最严格水资源管理在全省考核获优秀等次，饮用水源水质保持100%达标，广佛跨界河流水质改善取得新突破。划定黑烟车限行区，淘汰黄标车及老旧车1.7万辆，完成国家大气污染防治终期考核目标。全面落实中央环保督察整改，基本完成100项环保民生实事，完成原定禁养区畜禽养殖场关停清理，自然村人居生态环境综合整治完成率92.5%。开展村级工业园区“一村一策”环境整治提升，关停企业1420家，整治提升2979家。城市管理精细化水平提高。落实城市管理精细化行动计划，推进城市执法体制改革。加大城市管理考评力度，推行考评区域划分和季度全覆盖。完善数字城管信息采集制度，建立建筑垃圾、环卫车辆智能管理平台。城市治理智慧化水平居全国第8名［9］。

　　六是深入推进改革开放合作。各领域改革扎实推进。出台国家制造业转型升级综合改革试点三年行动计划。顺德区划定全国首个产业发展保护区，南海区成为省深化投融资体制改革试点和创建省社会信用体系建设示范点。推进中国（广东）国际贸易“单一窗口”建设，“单一窗口”国家标准版实现口岸全覆盖。深化国资国企改革，完成市属国有企业改革重组，佛山燃气集团上市。改革大数据统筹发展体制，组建全省首个数字政府建设管理局。“放管服”改革力度加大，开展“减证便民”行动，“一门式一网式”政府服务改革“佛山模式”入选国家“互联网+政务服务”示范工程。发出全省首张金融功能电子营业执照。实施商事主体住所登记管理暂行办法，新登记市场主体12万户，增长40.1%。总体完成农村土地承包经营权确权，颁证率98%。开放型经济新体制加快构建。新增境外直接投资企业43家，协议投资额46.2亿美元，增长5.7倍。碧桂园、联塑、国星光电、东方精工等骨干企业加快多元化海外投资并购步伐，美的收购德国库卡、打造美的库卡智能科技园区。在匈牙利、澳大利亚设立佛山泛家居品牌产品展示体验馆，中德工业城市联盟成员扩大至33家。积极融入粤港澳大湾区建设，与广州、中山共建南沙港，广中江高速二期等建成通车。构建“佛山+香港”合作新模式，达成7大领域11个重点项目合作意向。加快建设粤桂黔高铁经济带合作试验区（广东园），与贵阳、南宁等地高新区签署合作协议。与延安、营口缔结友好城市，与俄罗斯纳罗福明斯克区、瓦努阿图维拉港市结为国际友好城市。落实27.5亿元财政资金，对口支援西藏墨脱县和新疆伽师县、新疆生产建设兵团农三师草湖产业园，对口扶贫协作四川凉山州，支援联系四川甘孜州乡城县和得荣县，对口合作黑龙江双鸭山市工作积极推进，新时期精准扶贫对口帮扶湛江、云浮深入开展，佛山云浮氢能产业共建取得重要成果。

　　七是加快发展社会民生事业。全市民生支出573.5亿元，占一般公共预算支出73.9%。省、市民生实事圆满完成，在省基本公共服务均等化绩效考评中再获第1名。基本公共服务水平稳步提升。城镇新增就业8.6万人。全市人社系统发放创业担保贷款1.7亿元，带动4.2万人创业就业。最低生活保障标准提高到每人每月900元，城乡居保基础养老金标准上调至180元。推进基本医疗保险城乡一体化改革，实现职工和居民待遇标准统一，基本医保参保511万人，增加10万人。全面提升教育现代化水平，推进基础教育强师工程和普通高中优质多样特色发展，实施义务教育阶段学校基础设施五年提升行动计划，新（改、扩）建各类义务教育学校33所，新增学位3.3万个。建设卫生强市，推进城市公立医院综合改革，全面取消药品和医用耗材加成；　完善分级诊疗制度，三级公立医院均参加医联体［10］建设；新注册登记执业医疗机构248家，其中非公立医疗机构225家。棚户区改造开工1069套，基本建成285套；　公租房基本建成1105套，分配4426套。加强新市民服务，启用“互联网+新市民”自助申报平台，新增户籍人口19.4万人。蝉联全国文明城市。出台文化佛山三年行动计划和文化产业融合发展实施意见，通过创建国家公共文化服务体系示范区中期督查，禅城、南海、顺德区创建成为首批省公共文化服务体系示范区。第九届市运会、秋色巡游、创意城市博览会、香港·佛山节、功夫（动作）电影周、50公里徒步等活动成功举办，中国南方影视中心、岭南方言文化博物馆启动建设，佛山文化馆新馆、全民健身活动中心建成开放。平安佛山建设深入推进。社会治安防控体系建设和“飓风2017”专项打击整治行动成效位居全省前列，110接报刑事警情下降38.5%，社会治安稳定向好。创建国家食品安全示范城市扎实推进，累计建成省市级食品安全示范街19条、食品小作坊集中加工中心15个，食品抽检合格率97.4%，连续3年未发生较大以上食品药品安全事故。粮食安全责任省年度考核成绩优秀。构建“一系统”“一图纸”“一表格”［11］的城市安全风险管控格局，全面深入开展安全生产大检查，在全省率先出台安全生产领域改革发展实施意见，较大以上生产安全事故防控成效明显，生产安全事故总量下降5.8%。

　　八是着力建设人民满意政府。认真学习贯彻党的十九大精神。坚持向市人大及其常委会报告工作、向市政协通报情况，自觉接受监督，办理人大代表建议168件、政协提案209件，办复率100%。提请市人大常委会审议地方性法规草案1项，制定政府规章3项。推进法治政府示范区建设。强化科学民主决策，组建市政府立法顾问专家库、市重大行政决策咨询论证专家库。加快政府职能转变，完成承担行政职能事业单位改革试点任务，实现市、区、镇（街道）三级权责清单动态监管。深化政务公开，连续7年获全国地级市政府网站绩效评估第1名。完善督查制度，对各区开展大督查。全面落实行政执法责任制，推动审计制度改革，强化审计监督。加强党风廉政建设，落实重大工程廉洁风险同步预防制度。严格落实中央八项规定精神，严厉整治“四风”，治理“为官不为”，政府作风持续改善。建设人民满意政府行动完成128个年度重点项目，在全省反腐败满意指数和廉洁感知指数评比中名列前茅［12］。

　　各位代表，过去一年取得的成绩来之不易。这是习近平新时代中国特色社会主义思想正确指导的结果，是党中央、国务院，省委、省政府和市委正确领导的结果，是全市人民团结奋斗的结果。在此，我代表市人民政府，向全市人民，向各位人大代表、政协委员，向各民主党派、各人民团体、各界人士，向中央、省驻佛山有关单位、人民解放军、武警部队官兵，致以崇高的敬意！向长期关心支持佛山改革发展和现代化建设的港澳台同胞、海外侨胞及国际友人，表示衷心的感谢！

　　我们也清醒地看到，经济社会发展中还存在不少困难和问题，主要是：发展不平衡不充分的一些突出问题尚未解决，发展质量和效益有待提升，制造业总体层次和水平还不够高，自主创新能力不够强，高端创新平台不足，地方政府债务特别是隐性债务风险不容忽视，金融支持实体经济有待强化，现代服务业发展不充分；生态环境保护任重道远，土地开发强度偏高，大气、水环境治理仍需持续发力；城市形态功能品质有待优化，城市综合管理水平有待提升；全市统筹协调工作机制有待完善，区域发展不平衡、城乡发展不协调问题仍然存在，民生领域还有不少短板，优质公共服务供给有待加强；安全生产形势依然严峻，企业主体责任仍未得到有效落实；少数政府工作人员干事创业精气神不强、能力不足、责任担当意识不够，工作作风亟待转变。我们一定要直面挑战、迎难而上，全力以赴做好政府工作，不辱历史使命，不负人民重托。

2018年工作安排

　　2018年是贯彻党的十九大精神的开局之年，是改革开放40周年，是决胜全面建成小康社会、实施“十三五”规划承上启下的关键一年。从国际看，经济格局、增长动能、治理体系正发生深刻转变，尽管仍存在不确定性，但贸易和投资回暖，金融市场预期向好，世界经济复苏态势明朗。从国内看，经济正由高速增长阶段转向高质量发展阶段，处于转变发展方式、优化经济结构、转换增长动力的攻关期，虽然发展不平衡不充分问题仍然突出，但经济回稳向好的基本面并未改变。从佛山看，发展尽管面临不少压力和挑战，但依托雄厚的制造业实力、活跃的民营经济以及良好的市场环境，经济增长的基础较好、潜力较大、动力较足。总的来说，佛山正处于一个大有可为的历史机遇期，将有力推动佛山在更高起点上实现更高质量、更有效率的发展。只要我们坚定发展信心、保持战略定力，乘势而上、主动作为，认真做好全年各项工作，就一定能展现新时代的新气象、新作为，奋力开创佛山经济社会发展新局面。

　　今年政府工作指导思想是：在以习近平同志为核心的党中央坚强领导下，坚持以习近平新时代中国特色社会主义思想为指引，全面贯彻落实党的十九大精神和习近平总书记对广东工作重要指示批示精神，按照省委、省政府决策部署和市委十二届五次全会部署，坚持稳中求进工作总基调，坚持新发展理念，紧扣社会主要矛盾变化，按照高质量发展的要求，统筹推进“五位一体”总体布局和协调推进“四个全面”战略布局，以供给侧结构性改革为主线，突出创新驱动，深化改革开放，推动质量变革、效率变革、动力变革，在打好防范化解重大风险、精准脱贫、污染防治三大攻坚战方面取得扎实进展，引导和稳定预期，加强和改善民生，促进经济社会持续健康发展。

　　今年经济社会发展主要预期目标是：地区生产总值增长8%左右；全社会固定资产投资增长15%；社会消费品零售总额增长10%；进出口总额增长3%；地方一般公共预算收入增长9.5%；研发经费支出占地区生产总值比重达2.8%；居民消费价格涨幅控制在3%以内；居民人均可支配收入增长与经济增长基本同步；城镇新增就业7.8万人，城镇登记失业率控制在3.5%以内；单位地区生产总值能耗、主要污染物排放量下降幅度完成省下达的目标任务。

　　为实现上述目标，我们将重点抓好十方面工作：

　　一、坚定不移深化供给侧结构性改革，促进产业迈向中高端，建设现代化经济体系。以深化供给侧结构性改革为主线，把发展经济的着力点放到振兴实体经济上来，重点在“破、立、降”上下功夫，推动佛山制造向佛山创造、佛山速度向佛山质量、制造大市向制造强市转变。

　　着力推进“破、立、降”。大力破除无效供给，妥善处置“僵尸企业”，推进国际产能合作由注重产品输出转向注重标准和品牌输出。加快培育新动能，强化科技创新，加快发展先进制造业、战略性新兴产业和现代服务业，在中高端消费、创新引领、绿色低碳、现代供应链等领域加快形成经济新增长点。把支持企业做优做强做大与培育“专精特新”中小企业结合起来，形成大中小微企业专业化分工协作的产业生态体系。壮大一批骨干企业和领军企业，年主营业务收入超百亿元企业增至20家。落实“四上”企业培育三年行动计划，净增1100家“四上”企业，总数达1.21万家。培育一批细分领域“单打冠军”，新认定细分行业龙头企业200家。综合施策降成本，全面落实“粤十条”［13］“佛十条”，支持传统行业搭建产业链整合服务平台，着力降低企业生产要素成本、制度性交易成本等，打造制造业成本“洼地”。

　　加快建设制造强市。制造业是实体经济的主体，是供给侧结构性改革的重要领域。培育壮大新兴产业。推动电子信息、生物医药、新材料等新兴产业跨越发展，用好电子信息产业基金，引进集成电路、新型传感器、新型显示等引领型项目，抢占产业发展制高点。优化升级传统产业。实施新一轮技术改造，力争再用3年时间，全面完成规模以上工业企业开展技术改造。支持企业瞄准国际标准引入新技术、新管理、新模式，全面提高产品技术、工艺装备、能效环保水平，推动传统产业焕发新活力。大力推进智能制造。加快发展增材制造、高性能医疗器械、智能制造装备与自动化控制系统等高端装备制造，开展数字化车间、智能工厂试点，新增市级“中国制造2025”试点示范企业20家、智能化技改示范企业30家、机器人应用3000台。推动互联网、大数据、人工智能与制造业深度融合，发展壮大数字经济，积极培育网络化协同、个性化定制、在线增值服务、分享制造等“互联网+制造业”新模式，促进制造业向数字化、网络化、智能化发展。推动先进制造业集群化发展。坚持立足前沿、面向未来，加快智能家电、机器人、新能源汽车等产业集群化发展，力争通过若干年努力，打造若干个世界级先进制造业集群，促进产业迈向价值链中高端。突出以科技创新为导向的招商引资，办好珠江西岸先进装备制造业投资贸易洽谈会、中国（广东）国际“互联网+”博览会等重大展会以及中国制造论坛等重大活动，引进一批技术含量高、质量效益好、带动能力强的重大项目，为经济发展增添新动力。

　　促进制造与服务协同发展。大力发展研发设计、现代物流、电子商务等生产性服务业，推动生产性服务业向专业化和价值链高端迈进。引导制造业企业从主要提供产品向提供产品和服务转变，加快服务环节专业化分离和外包，提高制造业附加值和竞争力。打造“工业设计之都”，发挥广东工业设计城龙头作用，培育一批具有核心竞争力的设计机构、国际知名的工业设计大师和有世界影响力的设计品牌。

　　实施智慧物流腾飞计划，提升快递物流、供应链金融等服务功能，推动现代物流向供应链集成延伸。加快发展商贸会展业，发挥广东潭洲国际会展中心等平台作用，推进商贸会展与优势产业融合发展，提升会展品牌化专业化运营水平。加快发展健康养老、旅游休闲、教育文化等生活性服务业，促进生活性服务业向精细化和高品质转变。发挥消费对经济发展的基础性作用，完善促进消费的体制机制，实施放心消费创建工程，大力发展信息消费、绿色消费等新业态，优化城市商圈规划建设，引导商业综合体特色化错位发展，提升消费环境和品质。

　　全面提高供给体系质量。继续实施“以质取胜、技术标准、品牌带动”战略。牢固树立“质量第一”意识，积极创建全国质量强市示范城市，落实工业产品质量提升行动计划，提高产品和服务质量，显著增强佛山质量优势。建设先进标准体系，以高标准引领高质量，新制定联盟标准数量15项以上，主导或参与制（修）订各级标准20项以上，推动科技创新成果转化为技术标准20项以上。加强个体品牌和区域品牌建设，力争省名牌产品数量不少于530个，继续保持全省首位，确保全国知名品牌示范区数量稳居全国地级市首位。弘扬劳模精神和工匠精神，培育聚集更多能工巧匠、大城工匠，建设现代职业教育综合改革示范市，不断壮大知识型、技能型、创新型劳动者队伍。

　　二、深入实施创新驱动发展战略，提高科技创新能力，加快建设面向全球的国家制造业创新中心。将创新贯穿于产业发展的全领域、全过程，加强创新要素集聚和科技成果转化，加快形成以创新为主要引领和支撑的现代化经济体系和发展模式。

　　加强科技创新能力建设。加强基础研究和应用基础研究。坚持科技创新面向世界科技前沿、面向经济主战场、面向国家重大需求，高起点、高标准建设先进制造科学与技术广东省实验室，加大关键共性技术、前沿引领技术、颠覆性技术创新攻关力度。强化企业自主创新主体作用。推动高新技术企业数量扩张和质量提升并举，力争国家高新技术企业达3500家以上，培育一批具有创新能力的排头兵企业，推动更多中小型高新技术企业上规模。支持大型企业承担参与国家和省重大创新平台建设，引导中小企业建立研发机构，规模以上工业企业研发机构建有率提高到50%。提升科技公共服务水平。加快建设广东高校科技成果转化中心，做强做大中科院佛山产业技术研究院、佛山智能装备研究院等一批新型研发机构，深化与中国科学院、中国工程院、清华大学等高水平科研院校合作，打造清华大学“一园一院一中心一基金”［14］政产学研合作模式。完善孵化育成体系，鼓励孵化平台载体做精专业、做优服务、做强品牌，引导龙头骨干企业延伸产业链建设一批“双创”平台，各类孵化载体增至65家，其中国家级孵化器16家、国家级众创空间20家。建设国家知识产权示范市。积极构建以企业为主体、市场为导向、产学研深度融合的技术创新体系，促进科技成果在佛山转移转化。

　　高水平规划建设区域创新平台。以超前理念，高起点规划、高标准建设禅南顺（三龙湾）高端创新集聚区，加快佛山军民融合创新示范区、南三产业合作区等平台规划建设，构建“一环创新圈”“1+5+N”创新平台体系［15］，积极对接广深科技创新走廊，主动承接深圳创新溢出效应。推动广佛创新驱动同城，广佛同心共同融入全球创新网络，促进南海区青年湖电子信息产业园与广州白云区黄金围产业园联动发展。强化佛山国家高新区在全市创新发展中的核心带动作用，实施“聚焦高新629”［16］计划，建设科技创新小镇群，争创全国高新区20强。

　　推进军民融合发展。把军民融合深度发展作为促进产业结构优化升级的重要引擎，主动对接军队高端科技创新资源和成果，积极构建军民融合协同创新体系，大力支持“民参军”，积极承接“军转民”，推动军事装备生产技术和研发成果率先在佛山转化。面向智能装备、智能穿戴、航空航天、新材料等前沿领域，建设广东国防科技工业技术成果产业化应用推广中心、佛山中国空间技术研究院创新中心、顺德区军民融合创新产业园等平台载体，加强与军事科学院等机构合作，积极引入军工企业和重大项目，加快集聚军地优势资源，发展军民融合产业集群，打造国家级军民融合创新示范区。

　　深化金融科技产业融合。发展壮大金融产业，优化融资结构，打造充分满足实体经济需求的金融链。完善财政投入稳定增长机制，建立投资主体多元化、投资渠道和投资方式多样化的政策支持体系，鼓励多种投资主体参与创新创业。加快发展多层次资本市场体系，力争新增上市企业10家以上、股份制改造企业40家以上。扩大政策性金融工具覆盖面，发展科技信贷和科技保险，完善中小微企业融资服务体系。提升广东金融高新区发展内涵，建设珠江西岸创投中心和融资租赁区域中心，全市股权投资机构增至400家、注册资本600亿元。

　　打造高水平创新创业人才队伍。坚持“三个舍得”［17］，加大高层次人才队伍建设投入，引进培育一批具有国际水平的科技领军人才、高技能人才和高水平创新团队，打造人才集聚高地、创新创业乐土。新增市级以上创新创业团队40个以上，新引进全职国家级和省级领军人才20名以上、地方领军人才30名以上。创新人才政策，在全国设立5个“人才驿站”，建设高层次人才“一站式”服务专区，落实住房、医疗、教育等配套政策，吸引更多高端人才来佛山安居乐业。打造南方高等教育名城，推进佛山科学技术学院创建高水平理工科大学，以全新体制机制筹建佛山理工大学，加快广东省研究生联合培养基地（佛山）及南方医科大学、广东财经大学两个全学段校区建设，确保北京科技大学、北京外国语大学两个研究生院秋季正式招生。

　　三、推动更高层次开放合作，拓展开放领域和空间，加快构建全面开放新格局。以全球视野和战略眼光谋划开放布局，推动佛山与周边城市乃至世界的经济联系更加紧密、相互合作更加深入，全面提高佛山经济国际竞争力。

　　积极参与“一带一路”建设。深化与“一带一路”沿线国家在经贸、科技、文化等领域合作交流，构建具有更强国际竞争力的开放型经济新体制。积极开展产能合作，引导更多企业到沿线国家投资设厂，鼓励优势行业通过并购或直接投资模式，实现全球化布局经营，提高产品和品牌知名度。拓展对外贸易，在美国、非洲等地建设3个以上佛山泛家居品牌产品海外展示体验馆，构建完善自主营销网络，扩大自主品牌产品出口。复制推广自贸试验区改革创新经验，提升贸易和投资自由化便利化水平。发展外贸综合服务企业和服务平台，争取市场采购试点落地，鼓励支持扩大优质消费品、先进技术装备等进口。扩大利用外资规模，深化对德对欧合作，推进中德工业服务区建设，扩大中德工业城市联盟影响力，加强与友好城市交流，拓展佛山国际“朋友圈”。

　　主动融入粤港澳大湾区建设。全面对接广州、深圳、香港等中心城市，建设粤港澳大湾区枢纽城市。以广佛同心携手打造珠三角世界级城市群核心为引领，落实广佛同城化发展规划，重点抓好广佛环线及对接广州的9条地铁线规划建设，推进荔湾—南海、番禺—顺德、花都—三水同城化合作示范区建设，深化两地在交通、产业、科技、民生、环境治理等方面的合作。以港珠澳大桥、深中通道、虎门二桥、广深港高铁等建设为契机，加强与深圳、东莞等城市在技术、资本、人才等创新要素上的对接，参与构建粤港澳大湾区协同创新体系。深化与香港在科技创新、现代服务业等领域合作交流，建设粤港澳合作高端服务示范区。融入广东省沿海经济带，打造珠江西岸先进装备制造产业带创新引擎、华南科技金融产业融合创新中心［18］，增强区域辐射带动能力。

　　推动更高水平区域合作发展。参与编制广佛肇清云韶经济圈发展总体规划，建设广佛肇经济合作区及广佛肇高速、佛清从高速等项目。依托佛山西站推动高铁经济带加快发展，加快粤桂黔高铁经济带合作试验区（广东园）建设，争取率先成为全国高铁经济带合作示范区。打好精准脱贫攻坚战，瞄准特定贫困群众精准帮扶，向深度贫困地区聚焦发力，激发贫困人口内生动力。深入推进新一轮精准扶贫对口帮扶湛江、云浮工作，推进佛山云浮产业共建，加快“两园六区”［19］开发建设，完成“一区一龙头项目”建设任务。做好对口支援西藏墨脱县、新疆伽师县、新疆生产建设兵团农三师草湖产业园及支援联系四川甘孜州乡城县和得荣县工作，推进对口四川凉山州扶贫协作，开展与黑龙江省双鸭山市对口合作，在全国精准脱贫攻坚战中贡献佛山力量、体现佛山担当。

　　四、深化重点领域和关键环节改革，进一步完善社会主义市场经济体制，激发各类市场主体活力。以纪念改革开放40周年为契机，以完善产权制度和要素市场化配置为重点，深入推进经济体制改革，使市场在资源配置中起决定性作用，更好地发挥政府作用，增创体制机制新优势，释放经济社会发展新潜能。

　　重振改革精神。在改革开放40周年之际，总结经验、乘势而上，重振佛山改革创新精神。坚定不移做改革的闯将、实干家和促进派，保持锐意进取的勇气、敢为人先的锐气、蓬勃向上的朝气，勇于冲破思想“禁区”、利益“雷区”和束缚发展的体制藩篱，按照先行一步、走在前列的要求，不断创新改革思路和举措，逢山开路，遇水架桥，将改革进行到底。

　　优化提升营商环境。推进国家制造业转型升级综合改革试点，探索完善制造业发展体制机制。深化“放管服”改革，完善新型市场服务监管模式，加快转变政府职能，打造市场化法治化国际化营商环境。深化行政审批制度改革，取消调整一批行政许可事项、公共服务事项和行政审批中介服务事项，公布投资项目报建审批事项清单。完善“一门式一网式”政府服务模式改革，深化企业经营准入主题式服务和企业投资建设联合审批改革，推进跨部门、跨层级、跨区域通办。推动线上线下服务融合，拓展网上办事大厅、手机移动终端和“市民之窗”自助终端办事深度。深化商事制度改革，实现开办企业减环节、压时限、准入即准营，打造全市统一的商事登记系统。发挥工商联和各类商（协）会作用，畅通政企沟通渠道，规范政商交往行为，构建“亲”“清”新型政商关系。常态化开展暖企行动，落实领导挂点联系企业制度，帮助企业解决发展难题。

　　完善产权保护制度和要素市场化配置。以公平为核心原则，完善产权保护制度，依法保护各种所有制经济的产权和合法利益，严肃查处各类侵权行为，实现产权有效激励。培育和壮大企业家队伍，激发和保护企业家精神，使企业家安心经营、放心投资。强化各种所有制企业的市场主体地位，深化国有企业改革，发展混合所有制经济，支持民营企业跨越发展，加快外资企业“引进来”步伐。开展市场准入负面清单制度改革试点，清理废除妨碍统一市场和公平竞争的规定做法，破除歧视性限制和各种隐性障碍，激发各类市场主体活力。完善要素市场化配置，深化户籍、社会保障、土地等制度改革，放宽市场准入，整合优化公共资源交易平台，使市场在资源配置中起决定性作用。

　　深化行政管理体制改革。强化市级宏观调控能力，加强重大政策设计、重大项目布局和重点资源配置能力建设。强化区级经济发展和创新集聚职能，增强区级对中央、省、市的重大决策、重大规划、重大项目的落实力、执行力。强化镇（街道）公共服务、社会治理等职能，提高基层直接服务企业和群众的能力。以数字政府建设管理局成立为契机，推进政务信息系统整合共享和政务大数据库建设，加强大数据在政府决策、城市管理、社会治理、企业服务和民生事业等方面的运用。深化事业单位分类改革，巩固承担行政职能事业单位改革成果，推进从事生产经营活动事业单位改革，优化事业单位管理体制和运行机制。

　　深化国资国企改革。完善国有资产管理体制，创新监管方式，实现从管资产向管资本转变，提升国资监管能力和实效。优化国有资本布局结构和运营效率，引导国有资本重点投向基础设施、公共事业、重大平台和战略性新兴产业。积极推进混合所有制改革，以企业上市为突破口，引进民资、外资参与国企改制重组，推动国资做强做优。完善国企法人治理结构，健全灵活高效的市场化经营机制。

　　五、推动全市统筹协调发展，提升城市形态功能品质，加快建设高品质现代化国际化大城市。围绕城市发展中存在的城市形态与经济发展水平之间、城区与村镇之间、各区之间不平衡不充分问题，在协调上下功夫，在统筹上求突破，打造宜居宜业宜创新的城市环境。

　　推动全市区域统筹协调发展。牢固树立全市“一盘棋”理念，围绕基本公共服务均等化、基础设施通达程度比较均衡、人民生活水平大体相当的目标，加大市级在重大发展规划、重大基础设施、重大平台载体等领域统筹力度。推进“三规合一”［20］编制，理顺市、区、镇（街道）三级规划管理体制和职能，推动全市控制性详细规划全覆盖。着力构建全市统筹规划、各区协调联动发展新格局，研究建立全市各区统筹联动议事决策工作机制，加强各区土地开发规划、招商引资、重大项目、生态环境、公共服务等重大事项对接。深入实施“强中心”战略，将禅城、南海、顺德区打造成为广佛都市圈核心区。促进各区竞合发展，发挥禅城区的中心城区辐射带动作用，南海、顺德区的区域合作主力军作用，引领全市实现更高水平、更高质量发展；发挥高明、三水区后发优势，推动产业资源、基础设施、公共服务等适度向高明、三水区倾斜，促进两区加快发展速度、优化经济结构、提升服务业发展水平，实现跨越式发展。

　　优化提升城市形态功能品质。实施中心城区城市形态提升专项行动，加强城市设计和村庄规划，从整体平面和立体空间上统筹城乡建筑布局，推动重要通道沿线、城中村等区域面貌总体改善，实现“一年一小变、两年一中变、三年一大变”。建立城市更新常态化管理机制，组建城市更新局，落实“三旧”改造产业用地、公益性用地等政策，划定产业保护红线，提升城市品质和投资环境。加大村级工业园区整治改造力度，制定环境保护、安全生产等标准，明确功能定位，稳步推进连片整合开发、一体化规范化管理，将一批主题鲜明、配套完善的园区纳入重点改造和推广范围，示范引领全市村级工业园区改造提升。坚持以高水平规划引领城市现代化，落实城市治理三年行动计划，优化“强中心、多组团”网络型城市空间布局，推动城市中轴线、东平河一河两岸等区域品质整体提升，打造城市客厅及城市地标，营造高质量人居环境。积极推进首批市级特色小镇建设，谋划创建第二批市级特色小镇。

　　加强城市现代化基础设施建设。加快轨道交通建设。推动全市轨道交通“四网合一”［21］，开展轨道交通线网规划修编，加快地铁2号线一期、3号线等项目建设，做好2号线二期、4号线一期、9号线一期、11号线和13号线一期等前期工作，加强轨道交通站点TOD综合开发［22］。优化城市路网结构。加快“一环”高速化改造，实现主线贯通。推进“一环”西拓工程等项目建设，构建“两环四纵五横”高速公路网［23］和“八横七纵”快速路网体系［24］。实施“断头路”连通五年行动计划，新打通20条“断头路”。创建国家公交都市示范城市，深化公交TC改革［25］，加强公交枢纽、首末站建设，拓展公交专用道建设成网，规范共享单车发展。加快佛山沙堤机场迁建，力争年底前珠三角新干线机场先期工程动工，规划建设临空经济区，打造粤港澳大湾区西部航空枢纽。整合开发西江、北江岸线资源，优化港口规划布局，推进珠江黄金水道建设，加快发展临港经济区。完善通信、水利、电力等基础设施，确保110千伏新联输变电等工程投产。建设光网城市［26］和无线城市，扩大全市宽带网络覆盖面。建设海绵城市，各区完成总任务60%以上，全市新建地下综合管廊10公里。

　　提高城市管理精细化水平。将“精细化”贯穿城市规划建设管理全过程，管好管优中心城区，并加快向城乡结合部、镇村延伸拓展，努力实现全覆盖。加强城市数字化智慧化建设，完善全市统一的数字城管信息系统，促进交通、警务管理智能化。强化城市管理考评，提高市政设施、园林绿化的建设管养水平，推进道路“五位一体”［27］沿街景观提升。加大居民小区、村级工业园区等重点区域违法建设专项整治力度，坚决遏制增量，大力查处存量。完善城市管理法制体系，推进城市执法体制改革，推动执法重心下移和执法事项属地化管理。

　　实施乡村振兴战略。按照产业兴旺、生态宜居、乡风文明、治理有效、生活富裕的总要求，加快农业农村现代化，逐步实现城乡基础设施和公共服务一体化，走好城乡融合发展之路。推进农业供给侧结构性改革，扶持一批农业龙头企业、农民专业合作社和农村电子商务平台，发展高价值种植业、休闲农业等都市精品农业，以及田园综合体、民宿经济等新业态。推进农村集体产权制度改革，完成农村土地承包经营权确权登记颁证。全面推进美丽宜居乡村建设，建成首批110条美丽文明示范村居，继续推进6个高水平省级新农村示范片建设，“五好”［28］　新村居增至270个，改善农村居民生活条件。落实高明区革命老区乡村振兴三年特别帮扶计划。实施建设用地减量规划，推动空心村、空闲地等低效建设用地拆旧复垦。推进农村幸福社区建设，完善村（居）议事协商民主机制，加强村务监督委员会建设，深化村（居）务公开，健全自治、法治、德治相结合的乡村治理体系。

　　六、加强各类风险隐患排查，健全化解处置长效机制，打好防范化解重大风险攻坚战。增强忧患意识和底线思维，把防控风险放在更加突出位置，推进风险防控工作科学化、精细化，力争把各类风险化解在源头，筑牢经济发展安全防线。

　　严控政府债务风险。按照去存量、控增量的要求，进一步规范政府举债融资机制，促进债务结构优化，实现债务风险水平总体下降，预警地区全部脱离风险预警，保障财政平稳运行。严格落实政府债务限额管理要求，牢牢守住债务限额“天花板”，确保不突破债务限额。坚决遏制隐性债务增量，积极稳妥处置具有较高风险的隐性债务存量。落实债务风险防范化解工作计划，确保按期完成债务清理整改化解任务。

　　有效防范化解金融风险。完善地方金融风险监测防控平台，强化金融监管力量，加强对金融风险的监测预警和应急处置。推动金融机构简化产品结构、压降不良资产，加快金融去杠杆，加强流动性风险防范，促进各类金融机构相关指标达到监管标准，银行机构不良贷款率保持合理水平。严格规范实体经济企业从事金融业务，大力遏制资金“脱实向虚”。完成市级农村商业银行组建。严厉打击非法集资，整顿规范互联网金融，坚决守住不发生区域性系统性金融风险的底线。

　　促进房地产市场平稳健康发展。推进住房租赁试点和集体建设用地建设租赁住房试点，加快构建多主体供应、多渠道保障、租购并举的住房制度，促进房地产市场平稳健康发展。发展住房租赁市场特别是长期租赁，本着租购同权原则，保护租赁各方合法权益，培育和支持专业化、机构化住房租赁企业发展，到2020年推出10万套新建租赁住房。坚持“房子是用来住的，不是用来炒的”定位，持续开展房地产市场专项整治，坚决稳控市场预期，将房价维持在合理水平，加大非商品住房去库存力度，坚决防控房地产领域风险。

　　七、加快推进生态文明建设，打好污染防治攻坚战，用绿水青山塑造美丽佛山。牢固树立绿水青山就是金山银山、保护生态环境就是保护生产力的理念，标本兼治、铁腕治理，切实改善生态环境质量，探索工业城市绿色发展之路。

　　全面打好污染防治攻坚战。坚持全民共治、源头防治，在大气、水、土壤污染防治上精准发力、攻坚克难。重点打赢蓝天保卫战，实施大气污染防治行动，确保完成省下达的大气指标，PM2.5浓度明显下降。落实重点区域精准防控措施，推进70家省控重点企业“一企一策”综合整治，淘汰禁燃区内高污染燃料燃用设施，实施推广电能替代技术，减少机动车排气污染，严控工地扬尘、渣土车和非道路移动机械污染。实施水污染防治攻坚战行动计划，落实河长制和湖长制，加强重点流域综合整治，新建300公里污水管网，推进雨污分流，提高污水处理厂进水浓度并实施提标改造，加快农村分散式污水处理装置建设，完成新扩禁养区畜禽养殖关停清理，推进建成区黑臭水体实现长治久清，确保广佛跨界河流水质达到省考核要求。开展土壤污染调查和风险管控，加强生活垃圾处置，加快顺控环投热电项目建设，研究推进生活垃圾分类分流减量。强化固体废弃物处置，加快建设南海、顺德区绿色工业服务中心及相关配套项目。推进排污许可全覆盖，深化固定污染源环境管理制度改革。细化量化环保考核指标，强化环境执法，构建政府为主导、企业为主体、社会组织和公众共同参与的环境治理体系。

　　加强生态系统保护修复。建设大湾区高品质森林城市，开展大规模国土绿化行动，构建“三屏六楔、两脉两环、蓝绿成网”［29］的自然生态格局，实现森林围城、绿色进城。实施绿地建设行动计划，通过“三旧”改造尤其是村级工业园区整治按比例增绿复绿，推进退耕还林、退桉提质、立体绿化等重点生态工程，创建国家生态公园，新建（扩容）18个千亩以上生态绿心［30］，到2022年市域森林覆盖率提升至39.33%。提高城市公园、森林公园、湿地公园、生态公园等绿地覆盖密度，三年新建省级湿地公园4个、市区级湿地公园25个、镇级公园32个，实现建成区绿化覆盖率43％、人均公园绿地面积16.95平方米。高水平建设“一环生态圈”，严格保护控制48个河心岛的开发建设，建设交通干线生态廊道和滨水河道生态廊道，构建点、线、面互联互通的生态绿化格局。

　　推进绿色低碳循环发展。创建国家绿色发展示范先行区，落实主体功能区布局要求，在重点生态功能区、生态环境敏感区和脆弱区划定生态保护红线。健全自然资源资产产权制度，研究建立市场化、多元化生态补偿机制。改革生态环境监管体制，严格执行自然资源离任审计制度。构建绿色技术创新体系，发展氢能源、分布式光伏、节能环保等产业，建设中国标准化研究院绿色制造技术与标准创新研发中心，以及环保科技小镇、南海区国家环境服务业华南集聚区、顺德区环保科技产业园，培育一批绿色工厂和绿色制造示范企业。推广应用绿色能源，扩大充电桩及加氢站建设覆盖范围，推广氢能源在公交、物流等运输车辆中的应用。推进绿色建筑扩面提质，绿色建筑面积增至1000万平方米。申报国家装配式建筑示范城市，到2020年装配式建筑占新建建筑20%以上。倡导简约适度、绿色低碳生活方式，开展创建节约型机关、绿色社区及绿色出行等行动。

　　八、加快文化事业产业发展，弘扬城市人文精神，彰显国家历史文化名城内涵。坚持经济强市和文化强市并举，以高度的文化自信促进文化的繁荣兴盛，打造人文与繁华并茂、精神与物质共享的文化导向型城市。

　　加快文化事业发展。创建国家公共文化服务体系示范区，开展“佛山韵律”系列文化艺术活动，加快佛山博物馆、佛山大剧院、粤剧文化园等重点公共文化设施建设，实现基层综合性文化服务中心全覆盖，构建覆盖城乡、保基本、促公平的现代公共文化服务体系。实施城市文化推广计划，打造“世界功夫之城”“世界美食之都”“陶艺之都”“博物馆之城”等文化名片，树立大城品质、塑造大城形象、彰显大城气度。加强文物保护利用和文化遗产保护传承，加快东华里古建筑群修缮和梁园、康园建设，开展秋色巡游、“行通济”等民俗活动，争取举办泛珠三角非遗活动。开展全民健身活动，举办50公里徒步、亚洲龙舟锦标赛等赛事，备战省第十五届运动会。加强社区体育公园建设，佛山国际体育文化演艺馆投入使用。

　　提高文化产业发展水平。打造国艺影视城、珠宝产业园等文化产业集聚区，支持石湾陶谷创建国家级文化产业示范园区，推动文化创意、影视传媒、珠宝玉器等产业做强做大。扎实推进中国南方影视中心建设，打造一批影视产业基地和主题乐园，促进影视产业集聚发展。办好第27届金鸡百花电影节，举办创意设计展、陶瓷艺术周等文化会展活动，打造文化产业新品牌。促进文化资源与文化产业有机结合，创意设计与生产制造深度融合，提升工业品、消费品的文化内涵和附加值。实施“鲲鹏展翅”“百强文化企业”扶持计划，设立文化产业投资基金，发展体育动漫、电子竞技、低空航空运动等文化消费新业态，建设“岭南体验、品质生活”文化消费城市。

　　弘扬城市人文精神。积极培育践行社会主义核心价值观，以蝉联全国文明城市为契机，以更高标准、更高要求持续推进文明创建，深入实施公民道德建设工程，擦亮“大爱佛山”“信用佛山”“乐善之城”“敬业之城”“志愿者之城”等文明创建品牌。从细节着眼、实处着手，抓好“厕所革命”等城乡文明建设的具体工作，推动文明创建常态化、精细化，让文明成为美丽的城市风景。繁荣文化文艺创作，实施文艺精品锻造和文艺人才培育工程，围绕纪念改革开放40周年等重要时间节点，讴歌新时代、传播正能量，讲好佛山故事、唱响佛山好声音。

　　九、提高保障和改善民生水平，加强和创新社会治理，增强市民获得感、幸福感和安全感。坚持以人民为中心的发展思想，以造福人民为最大政绩，想群众之所想，急群众之所急，不断满足人民群众对美好生活的向往。

　　持续增进民生福祉。优先发展教育事业，着力解决中小学生课外负担重、“择校热”等问题，解决好婴幼儿照护和儿童早期教育服务问题。加快普惠性学前教育学位规划建设，规范办园行为。推进义务教育优质均衡发展，加快义务教育阶段学校基础教育设施建设，推动普通高中优质多样特色发展，建设国家特殊教育改革实验区。坚持创业带动就业，建设创业孵化示范基地群，实施高校毕业生就业创业促进计划和就业创业精准扶贫行动。提高低保和特困人员供养标准，建设社会救助信息平台，建成市受助人员托养中心。建立多层次社会养老服务体系，完善养老服务设施规划布局，新增社区居家养老服务中心60家以上，新建收养性养老床位3800张以上，建设市级老年养护院和新福利机构。构建复退军人服务体系，推进双拥优抚安置工作。深化医药卫生体制综合改革，健全现代医院管理制度，推进公立医院药品和医用耗材供应链改革，探索住院医保费用按病组分值付费模式。建设卫生强市和健康佛山，落实分级诊疗制度，实现医联体全覆盖，社区卫生服务机构建设达标率100%。落实全面两孩政策配套措施。保障粮食安全，全市粮食保供稳价。深化户籍制度改革，完善积分服务政策体系，提升“互联网+新市民”自助服务水平，增强新市民融入佛山获得感。

　　打造共建共治共享的社会治理格局。充分调动社会各方力量参与社区治理体系建设，健全网格化管理，推动社会治理重心下移，完善非户籍常住人口参与基层治理机制，提高社会治理社会化、法治化、智能化、专业化水平。充分发挥社会组织作用，加强社工人才队伍和志愿服务组织建设。加强综治信访维稳工作，健全社会矛盾纠纷排查化解机制。完善覆盖城乡的公共法律服务体系，健全“一村（社区）一法律顾问、一专职调解员”机制，促进基层治理法治化。创建全国社会信用体系建设示范城市。

　　深入推进平安佛山建设。加强社会治安防控体系建设，严厉打击和惩治黄赌毒、盗抢骗等违法犯罪活动，建设平安佛山。创建国家食品安全示范城市，实施食品药品安全质量大提升计划，建成“明厨亮灶”餐饮单位2万家，餐饮服务食品安全A级单位、食品安全示范点、食品快速检验点、市级示范药店各1000家。坚守安全生产红线，严格落实安全生产责任制，强化安全风险分级管控和隐患排查治理双重预防，加强交通运输、危险化学品、消防、建筑施工、特种设备等重点行业领域安全生产专项整治，坚决遏制重特大事故发生。完善突发事件应急体系，推进应急指挥系统标准化建设。

　　认真办好十件民生实事。一是分配公租房1.95万套，棚户区改造开工892套、基本建成474套，完成省下达住房保障任务。二是新（改、扩）建义务教育学校41所，新增义务教育阶段学位4.2万个，其中公办学位3.6万个。三是城乡居保基础养老金提高至每人每月220元。四是全面开展新生儿疾病免费筛查。五是孤儿养育标准提高至每人每月2000元。六是新增公交专用道11.8公里、高品质辅助公交线路10条，新增（更新）公交车辆300辆。七是新增（改造）公园绿地面积67.6公顷。八是实施100项环保民生实事。九是完成新增10个古村落活化升级，具备农村形态的村（居）均达到“五好”标准，全面完成自然村人居生态环境综合整治。十是建设18项民生水务重点工程。

　　十、全面加强政府自身建设，持续改进工作作风，促进政府治理体系和治理能力现代化。坚持和加强党的全面领导，牢固树立“四个意识”，坚决维护以习近平同志为核心的党中央权威，自觉在思想上政治上行动上同党中央保持高度一致，以全面从严治党的实际成效开创政府工作新局面。

　　坚持依法全面履职。深入贯彻全面依法治国要求，建设法治政府，推进依法行政。加强重点领域政府立法，完善政府立法程序和公众参与政府立法机制，严格规范公正文明执法，推进行政复议规范化。加强政务公开。坚持科学决策、民主决策、依法决策，健全重大行政决策机制和程序。依法接受人大及其常委会的监督，自觉接受人民政协的民主监督，主动接受社会舆论监督，认真听取人大代表、政协委员、民主党派、工商联、无党派人士和各人民团体的意见。

　　加强党风廉政建设。认真落实中央八项规定精神及其实施细则，驰而不息纠正“四风”，强化对厉行节约、公务接待、职务消费等规定执行情况的常态化监督。加强对重大工程廉政监督，确保“四个安全”［31］。配合推进国家监察体制改革试点，建立市、区监察委员会，实现对行使公权力的公职人员监察全覆盖。加强廉政教育和廉政文化建设，深化廉政风险防控，构建不敢腐、不能腐、不想腐机制。

　　持续改进工作作风。坚持作风建设永远在路上，坚决整治表态多调门高、行动少落实差等形式主义、官僚主义问题，建设人民满意政府。严格控制“三公”经费，大力整治文山会海。坚持问题导向，加强调查研究，强化督查问责，严格执行工作责任制，实施政府绩效管理，强化年度目标任务考核，不驰于空想，不骛于虚声，一步一个脚印，踏踏实实干好工作。落实“三个区分开来”［32］，建立健全激励机制和容错纠错机制，给干事者鼓劲，为担当者撑腰，全面从严治理庸政懒政怠政。加强干部队伍建设，不断提高适应新时代、实现新目标、落实新部署的能力，努力打造忠诚、干净、担当的高素质专业化公务员队伍。

　　各位代表！党的十九大描绘了宏伟蓝图，指明了前进方向。千里之行、始于足下，大道至简、实干为要。让我们更加紧密地团结在以习近平同志为核心的党中央周围，以习近平新时代中国特色社会主义思想为指引，在省委、省政府和市委的正确领导下，不忘初心、牢记使命，凝心聚力、扎实苦干，为在新时代开启佛山发展新征程、谱写佛山发展新篇章而不懈奋斗！
